# Cecal bascule presenting as internal hernia

**DOI:** 10.1093/jscr/rjae366

**Published:** 2024-05-31

**Authors:** Jane Tian, Hannah English, Shubham Bhatia, Youssef Mourad, Martine A Louis, Norman Khan

**Affiliations:** Department of Surgery, Flushing Hospital Medical Center, 4500 Parsons Blvd, Flushing, NY 11355, United States; College of Osteopathic Medicine, New York Institute of Technology, 101 Northern Blvd, Glen Head, NY 11545, United States; Department of Surgery, Flushing Hospital Medical Center, 4500 Parsons Blvd, Flushing, NY 11355, United States; Department of Surgery, Flushing Hospital Medical Center, 4500 Parsons Blvd, Flushing, NY 11355, United States; Department of Surgery, Flushing Hospital Medical Center, 4500 Parsons Blvd, Flushing, NY 11355, United States; Department of Surgery, Flushing Hospital Medical Center, 4500 Parsons Blvd, Flushing, NY 11355, United States

**Keywords:** cecal bascule, internal hernia, cecal volvulus, large bowel obstruction, virgin abdomen

## Abstract

Cecal bascule, a rare subtype of cecal volvulus, presents diagnostic and management challenges. We report a case of cecal bascule presenting as an internal hernia in a 68-year-old male with no surgical history. Computed tomography revealed two areas of mesenteric swirling and a displaced cecum. Prompt surgical intervention included laparoscopic exploration, resection of a necrotic adhesive band, and cecopexy. This case is noteworthy because of the absence of predisposing factors like prior surgeries or inflammatory conditions. Management options for cecal bascule include resection and cecopexy, tailored to individual patient factors. Awareness among healthcare providers is crucial for the timely recognition and appropriate management of such cases. Further research is needed to refine management strategies and improve outcomes for these rare but potentially life-threatening conditions.

## Introduction

Cecal volvulus predominantly affects females and younger patients in their second or third decade [1]. It presents either insidiously as a small bowel obstruction with abdominal pain, distention, and nausea/vomiting. Internal hernias are seen in 0.5%–4% of cases [2]. Prompt diagnosis and treatment prevent ischemia, necrosis, or perforation. Cecal bascule, a type of cecal volvulus, and internal hernia are rare causes of bowel obstruction, but have occasionally been described in combination in patients with a remote surgical history. We present a patient with a cecal bascule and an internal hernia due to an adhesive band in a virgin abdomen.

## Case presentation

**Figure 1:**
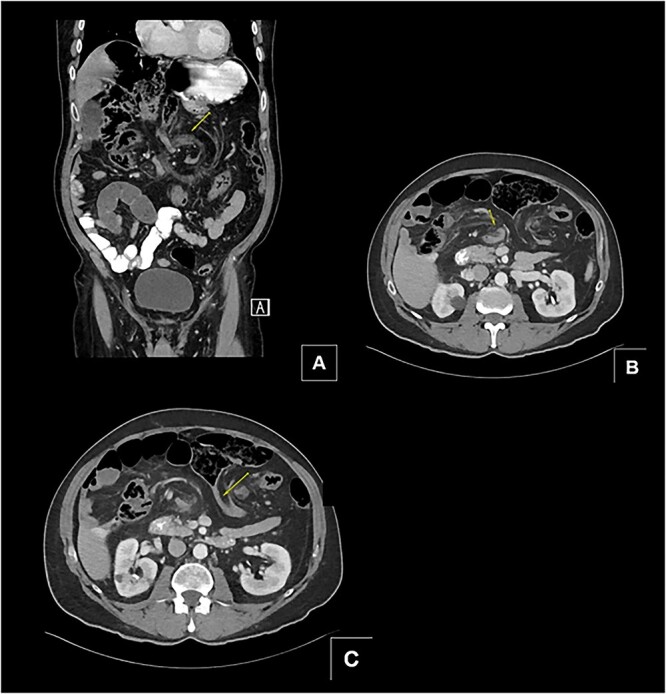
(A and B) Two separate CT images showing mesenteric swirling in distal small bowel and colon. (C) CT image showing severe luminal narrowing of distal transverse colon.

**Figure 2:**
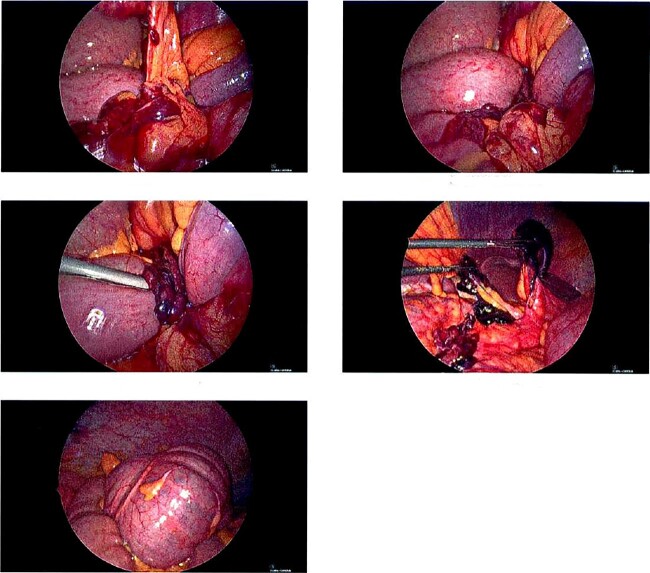
Intraoperative pictures of thick necrotic/adhesive band arising from colon mesentery entrapping band. Cecal bascule found in the RUQ.

A 68-year-old male with a history of hypertension and no past surgical intervention presented with 2 days of diffuse abdominal pain, anorexia, and nausea. On admission, his vitals included a temperature of 98.2°F, BP of 149/79, pulse of 64, and SpO2 of 97% with a mildly distended abdomen with tenderness in the umbilical and epigastric regions. The labs were unremarkable. CTA/P showed two separate areas of mesenteric swirling: one contiguous to the distal small bowel, resulting in small bowel obstruction, and another at the distal transverse colon (Fig. 1A–C). The cecum was noted to be in the right upper quadrant. The patient was emergently taken to the OR and underwent a diagnostic laparoscopy. A cecal bascule was noted in addition to an internal hernia caused by a thick adhesive necrotic band arising from the colonic mesentery and omentum (Fig. 2). The bowel was viable, and the band was resected. The transverse colon was tortuous, but no hernia was seen. The cecum was brought down to the right lower quadrant, and a cecopexy to the abdominal wall was performed. The patient had an unremarkable postoperative course and was discharged on postoperativhernia was seen. The cecum was brought down to the right lower quadrant, and a cecopexy to the abdominal wall was performed. The patient had an unremarkable postoperative course and was discharged on postoperative Day 2.

## Discussion

Cecal volvulus presents with twisting of the cecum, potentially causing obstruction and compromised blood flow. It is classified into three types. Type 1 and Type 2, representing 80% of occurrences, involve axial rotation with presentations in the right lower and left upper quadrants, respectively [1]. Type 3, known as cecal bascule, involves a hypermobile cecum folding anteriorly and superiorly on itself, usually in patients with abnormal cecal fixation [1–3]. Due to the lack of axial rotation of the colon, mesenteric vascular compromise is rare, and patients typically present with a less critical illness [4]. In these cases, ischemia would typically only occur from intraluminal tension or extraluminal compression from the borders of an internal hernia [3]. Clinical symptoms include abdominal pain, nausea, vomiting, and decreased bowel movements [5]. A vague clinical presentation such as this leads to a broad differential [1].

Imaging can aid in pinpointing the diagnosis, typically showing a displaced cecum to the right upper quadrant, a transition zone between the ascending colon and cecum, and/or perihepatic free fluid. Unlike type 1 and 2 cecal volvulus, the cecal bascule lacks the classic “whirl sign” on CT scans due to the absence of mesenteric twisting [3, 6].

Cecal bascule may arise from anatomical and clinical factors. Congenital or postoperative and post-inflammatory adhesions may cause fixation of the anterior cecal wall to the anterior wall of the ascending colon [2, 6]. Clinical conditions exacerbating bowel distension, such as distal mechanical obstruction, Ogilvie syndrome, postoperative ileus, and neurogenic bowel dysfunction, have also been identified as contributing factors [2, 4, 6]. The patient presented in this case had no history of abdominal surgery or pertinent medical history that could be attributed to an increased risk of developing a cecal bascule. This is what is referred to as a “virgin abdomen” meaning the patient had no prior surgery, radiotherapy, or known peritoneal inflammatory disease.

Internal hernias represent uncommon yet critical causes of intestinal obstruction. They occur within the abdominal cavity as viscera protrude through congenital or acquired orifices in the peritoneum or mesentery. Various types include paraduodenal, pericecal, foramen of Winslow, transmesenteric, transmesocolic, and transomental hernias [7, 8]. Omental band adhesions, often from previous surgeries or inflammation, can cause acute small-bowel obstruction. The formation of omental fibers leading to obstruction is usually predictable in patients with prior surgeries [9]. However, in a virgin abdomen, as in this case, it is rare.

This case is atypical: a cecal bascule presenting in a virgin abdomen, in the setting of an internal hernia due to an adhesive band. A handful of previous reports have described cecal bascules herniating through the foramen of Winslow or occurring in patients with prior abdominal surgery. The absence of known predisposing factors, such as previous surgeries or inflammatory conditions, makes this case particularly noteworthy.

Prompt surgical intervention is necessary upon diagnosis or suspicion of cecal bascule and/or internal hernia to relieve obstruction, restore bowel viability, and prevent recurrence. Treatment options include resection with or without ileostomy creation or detorsion and cecopexy, depending on bowel viability. Nonoperative strategies encompass methods, such as nasogastric and colonoscopic decompression, with some documented instances of success [10, 11]. Recurrence rates differ between management options. Right hemicolectomy is considered the gold standard due to its extremely low recurrence rate. However, for unstable or debilitated patients with nonperforated, viable bowel, cecopexy, and cecostomy tube placement may be considered, with recurrence rates ranging from 0% to 28% and mortality rates of up to 14% [12, 13].

Close follow-up is essential to monitor for any signs of recurrence or complications. Long-term outcomes depend on factors, such as the extent of bowel resection, the presence of underlying comorbidities, and the adequacy of surgical intervention. Patients may benefit from ongoing surveillance and preventive measures to minimize the risk of recurrence or the development of complications.

## Conclusion

Further research is warranted to better understand the pathophysiology, risk factors, and optimal management strategies for cecal bascule and internal hernias, particularly in patients with virgin abdomens. Advances in imaging modalities and surgical techniques may aid in early diagnosis, intervention, and improved outcomes for these rare but potentially life-threatening conditions. Additionally, education and awareness among healthcare providers are crucial to ensuring timely recognition and appropriate management of such cases.
